# Uncovering the cognitive processes underlying mental rotation: an eye-movement study

**DOI:** 10.1038/s41598-017-10683-6

**Published:** 2017-08-30

**Authors:** Jiguo Xue, Chunyong Li, Cheng Quan, Yiming Lu, Jingwei Yue, Chenggang Zhang

**Affiliations:** Department of Neurobiology, Beijing Institute of Radiation Medicine, State Key Laboratory of Proteomics, Cognitive and Mental Health Research Center, Beijing, 100850 China

## Abstract

Mental rotation is an important paradigm for spatial ability. Mental-rotation tasks are assumed to involve five or three sequential cognitive-processing states, though this has not been demonstrated experimentally. Here, we investigated how processing states alternate during mental-rotation tasks. Inference was carried out using an advanced statistical modelling and data-driven approach – a discriminative hidden Markov model (dHMM) trained using eye-movement data obtained from an experiment consisting of two different strategies: (I) mentally rotate the right-side figure to be aligned with the left-side figure and (II) mentally rotate the left-side figure to be aligned with the right-side figure. Eye movements were found to contain the necessary information for determining the processing strategy, and the dHMM that best fit our data segmented the mental-rotation process into three hidden states, which we termed *encoding and searching*, *comparison*, and *searching on one-side pair*. Additionally, we applied three classification methods, logistic regression, support vector model and dHMM, of which dHMM predicted the strategies with the highest accuracy (76.8%). Our study did confirm that there are differences in processing states between these two of mental-rotation strategies, and were consistent with the previous suggestion that mental rotation is discrete process that is accomplished in a piecemeal fashion.

## Introduction

Spatial abilities are important cognitive skills that are used in various everyday tasks, such as learning the environment, and during academic activities. How we determine that figure objects have the same shape despite differences in orientation or size is a common problem in the study of visual perception. Shepard and Metzler displayed projections of two unfamiliar three-dimensional (3D) figures and instructed subjects to determine whether the two figures were identical or not despite the differences in orientation^1^. Subjects commonly rotated one object clock-wise or counter clock-wise until it visually matches or mismatches the target object, and then make the decision^2^.

The most commonly accepted theory on how the cognitive system creates a mental representation of a visual stimulus is that representations emerge through a step-by-step process, in which subjects visually perceive individual segments of a stimulus and internalize the pieces to represent the whole stimulus, also known as the piecemeal strategy. The piecemeal strategy involves decomposing the stimulus figure into several pieces, mentally rotating one piece into congruence with the comparison figure, and then performing similar rotation of the other segments to confirm their parity. Eye fixation sequences during mental-rotation tasks also suggest a piecemeal strategy^3, 4^. As such, Just and Carpenter proposed that subjects first rotated one segment of the figure and later determined whether the other segments were rotated into congruence. Noton and Stark^5^ found that the internal representation is created by cognitively focusing on the angles or principle features of the visual stimulus and that when an initial stimulus is recognized and perceived through matching, a similar fixation pattern is employed on the matched stimulus; specifically, subjects fixate on the same lines, corners, or angles of the matched item in the same order as when encoding the prior stimulus.

Researchers have attempted to uncover the cognitive processes underlying mental rotation, but have not yet reached a conclusive answer^6^. Mental-rotation tasks are assumed to comprise five sequential cognitive-processing stages: (1) perceptual encoding of the stimulus, (2) identification of the stimulus and orientation, (3) mental rotation of the stimulus, (4) judgement of parity, and (5) response and execution^7, 8^. Nevertheless, Just and Carpenter^3^ identified component processes in a mental-rotation task by analysing the fixation paths of their subjects and by observing how these paths changed with angular disparity. The results suggested that three processing stages were involved: (1) search, (2) transformation and comparison, and (3) confirmation of a match or a mismatch between stimuli. Therefore, their interpretation mostly refers to stage 4 (judgement of parity) of the former processing model with elements of stage 3 (mental rotation of the stimulus) and, secondarily, to stage 2 (identification of stimulus orientation)^9^. Shepard and Metzler’s interpretation of mental rotation as a holistic cognitive process has been disputed in numerous studies ever since its introduction^10–15^. In mental-rotation tasks, the pattern of fixation shifts suggests piecemeal rotation^4^.

Mental rotation may be assumed to process through eye movements, as individuals’ fixations maintaining gaze on a single location^16^, are closely related to our ability to visually encode spatially distributed information^3, 17^. Eye-movement measurements provide a complementary approach to capture cognitive processes with high resolution. Eye-tracking technology offers the possibility of capturing visual behaviour information in real time and obtaining gaze position on specific stimuli^18, 19^. The obvious advantage of collecting fixation information is that the behaviour during each trial can potentially be deconstructed into various processing states whose durations can be directly measured. In addition, another advantage is that the rapidity of the fixation can match the rapidity of the processor to some extent. Eye-movement behaviour can be sampled at high frequencies (60 Hz~2000 Hz or higher); thus, the individual cognitive processes can be measured directly^3^.

Foundational experiments in this field demonstrated that eye-movement patterns of individuals are under cognitive control and are tailored to the task at hand^20, 21^. Subsequent investigations have shown that the sequence and duration of fixations are closely related to the specific target task^22–26^. For instance, Axel Larsen^15^ has suggested that visual performance in the classic mental-rotation paradigm of Shepard and Metzler may emerge through repeated execution of a mental rotation of one stimulus followed by a comparison of the transformed visual image with the other stimulus. In this work, eye-movement analyses supported key aspects of the model and showed that initial processing time was roughly constant until the first saccade switched between the stimulus objects, while the duration of the remaining trial increased approximately linearly as a function of angular disparity.

Markov and hidden Markov models (HMMs) have been applied in the fields of speech recognition (e.g., Rabiner^27^) and handwriting recognition (e.g., Nathan *et al*.^28^) extensively and successfully. To date, however, few researchers have utilized Markov models to study eye movements. The most common use of HMM in eye-movement research has appeared in analyses of the probabilities of transition from one area of information (AOI) to another^29–36^. This work has assumed that the fixation sequences are Markovian in character; that is, the probability of a transition from one fixation to another is independent on the prior fixation sequence^37^. As a time-series model, the HMM is therefore well suited for eye-movement data from mental rotation because it provides a more comprehensive description of the eye-movement patterns.

In both initial^3^ and recent studies^9, 38^, researchers have extracted features and constructed rules for classifying instances of the mental-rotation processing stages only by analysing the fixation switches of individuals instead of those of the studied populations. Moreover, these analyses of eye-movement data are often based on linear models or average parameters that fail to consider eye-movement sequence as time-series data and therefore do not account for variations within a task. To the best of our knowledge, no studies have attempted to classify mental-rotation processing stages by statistically analysing fixation sequence information. Additionally, no studies have attempted to differentiate between experimental variables in mental-rotation strategies.

## Purpose of the Study

In our current mental-rotation tasks, each block-pair figure was subdivided into a left- and right-side pair. Based on the piecemeal strategy in mental rotation proposed by Just and Carpenter^4^, it is inferred that subjects conduct mental rotation tasks with discrete processes, that is, subjects usually choose one-side pair as a reference figure consciously or unconsciously. Therefore, our goal was to investigate how processing changes as the subjects engage in two different strategies of mental rotation: (I) left-side-fixed right-side-rotated (LFRR), mentally rotate the right-side pair into alignment with the left-side pair; and (II) right-side-fixed left-side-rotated (RFLR), mentally rotate the left-side pair into alignment with the right-side pair. The current study contained three experimental sessions, self mentally rotate (SMR), left-side-fixed right-side-rotated (LFRR) and right-side-fixed left-side-rotated (RFLR). In the SMR task, subjects conducted mental rotation tasks with self-mentally-rotating strategy. We implemented experiments with data-driven modelling using a logistic regression, a support vector model (SVM) and a discriminative hidden Markov model (dHMM). To capture the relationship between mental-rotation processing and eye movements, we modelled the recorded time series of fixations and saccades by assuming potential states that are supposed indicators of the cognitive system switching between different processing states. We assumed that the statistical properties of the eye-movement patterns differed in each processing state. The best model topology (the number of hidden states) was identified by comparing several potential model topologies with cross-validation and choosing the one that best explained the unobserved data.

## Results

### Subjective reports

After finishing all the tests, the subjects were asked about some issues regarding the effectiveness of the experiment, namely, whether they implemented the experimental strategies in strict accordance with the experimental instructions or not (Scoring 1–5, 1 represents completely inconsistent with the instructions, 5 represents completely consistent with the instructions). All subjects reported their scores as 4 or 5, which we considered meeting the experimental requirements, at least in the subjective domain.

### Behavioural results

Data from three subjects were eliminated because their accuracy never exceeded 85%. In addition, data from two subjects were also unavailable due to inaccurate eye-movement calibration.

**Figure 1:**
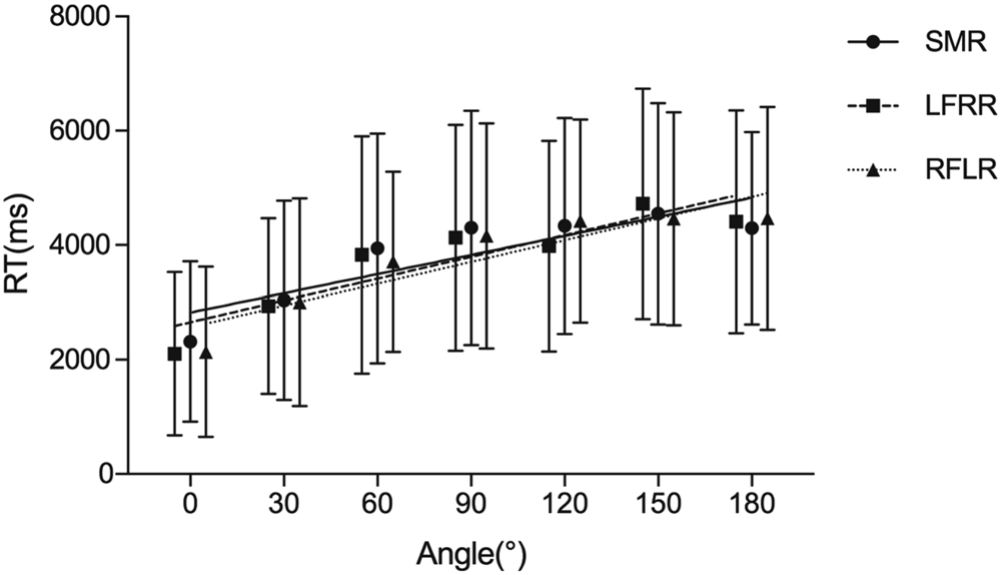
The RT of behavioural performance shows a strong angle effect. The grand average of RT increased almost linearly with the angular disparity. The amplitude of the angle effect on RT was similar between the LFRR task and the RFLR task. Error bars indicate standard errors of the means. The three lines indicate the linear regression trendlines.

The *angle effect* ^1^ of mental rotation was observed on both reaction time (RT) (*F*(5.304,222.768) = 94.589, *p* = 8.403E-55, *η* ^2^ = 0.387, *ε* = 0.884 and accuracy (*F*(4.800,201.600) = 9.184, *p* = 1.102E-7, *η* ^2^ = 0.084, *ε* = 0.800 indicating the successful completion of the three mental-rotation tasks^39^. RT increased almost linearly (Fig. 1), and accuracy correspondingly decreased with the angular disparity. ANOVA of RT and accuracy did not reveal a significant main effect of strategies (RT: *F*(1,28) = 0.089, *p* = 0.768, *η* ^2^ = 0.001; accuracy: *F*(1,28) = 0.016, *p* = 0.901, *η* ^2^ = 9.536E-5 indicating that behavioural performance was comparable when employing different mental-rotation strategies. The effect of the Strategy × Angle interaction showed a significant effect for RT (*F*(5.022,140.613) = 1.313, *p* = 0.262, *η* ^2^ = 0.015, *ε* = 0.837) and accuracy (*F*(4.843,135.607) = 0.929, *p* = 0.462, *η* ^2^ = 0.014, *ε* = 0.807). The *angle effect* of the two strategies was quantified by linear regression and compared using analysis of covariance (ANCOVA). The *angle effect* of the two strategies did not approach a significant effect (LFRR: 13.340 ms/degree, RFLR: 11.377 ms/degree, *F*(1,10) = 23.01, *p* = 0.075), which indicated almost similar behavioural performance on the two different tasks using different processing strategies. The session order effect was not significant for both RT (*F*(1,28) = 3.474, *p* = 0.073, *η* ^2^ = 0.008) and accuracy (*F*(1,28) = 0.422, *p* = 0.521, *η* ^2^ = 8.576E-4).

### Eye-movement results

The modelling dataset consisted of 2240 eye-movement sequences (20 subjects, each subject had two sessions using two different strategies – LFRR and RFLR – and each session consisted of 56 trials; 20 × 2 × 56) captured by an eye tracker. After eliminating invalid data from five subjects, the remaining dataset consisted of 1680 sequences, which were randomly divided into a training dataset of 1344 sequences and a test dataset of 336 sequences.

### Probability distributions of fixations and saccades

**Figure 2:**
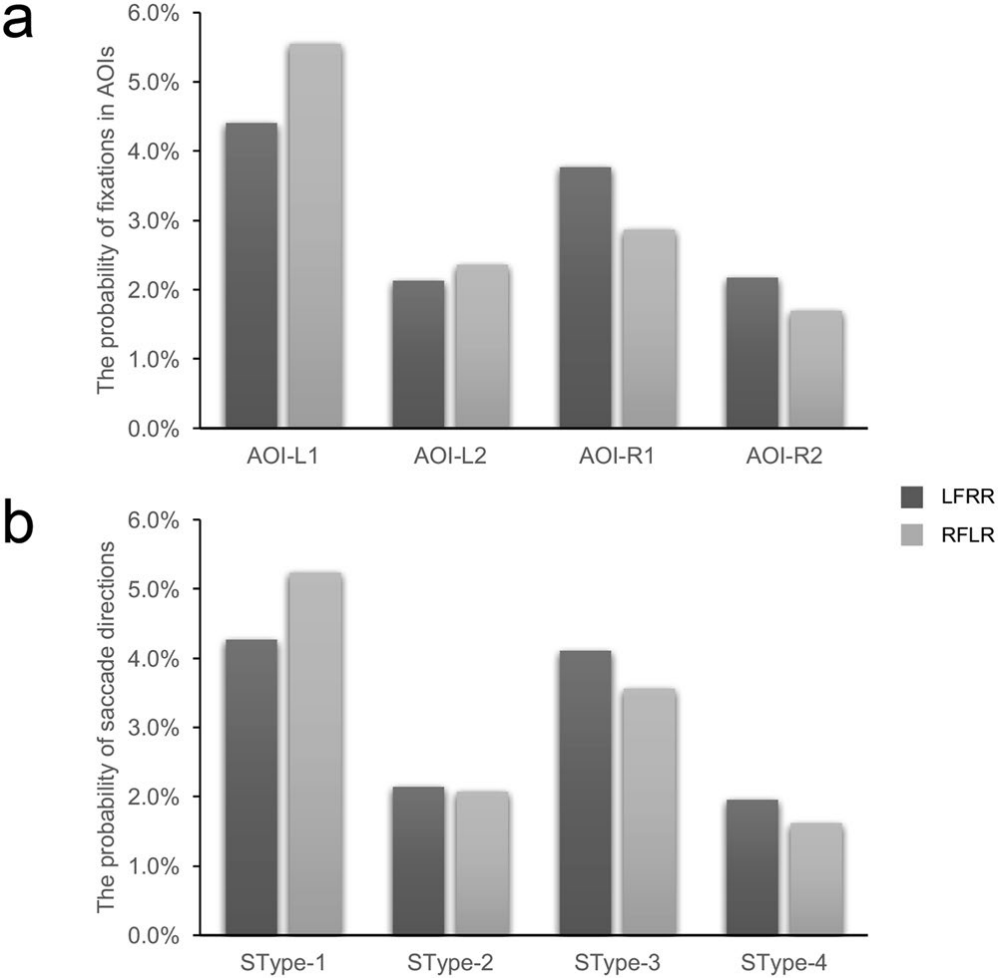
Probability distributions of the fixations and saccade directions. (**a**) The probabilities of fixations distributed on the four defined AOIs. (**b**) The probabilities of the four defined types of saccade directions. The blue histograms represent the values of the LFRR tasks, and the orange histograms represent the values of the RFLR tasks.

Before considering the three modelling classification approaches, we first conducted Wilcoxon rank sum test on the probability distributions of the fixations in different AOIs and the types of saccade directions to determine whether the fixations and saccades differed significantly respectively. The results were negative (fixations: *p* = 0.982; saccades: *p* > 0.999), suggesting that the distributions of fixations and saccades did not distinguish between or classify the mental-rotation strategies of the subjects (Fig. 2).

### Classification accuracy of the models

In the current study, the ground truth (the strategies of each subject) for a given fixation sequence was always known; therefore, the models based on this type of data belong to the general category of discriminative or supervised models, while the basic and simplest discriminative model is logistic regression^40^. Therefore, logistic regression was used as a simple classification model to obtain baseline results for the HMM. The confusion matrix of the logistic regression is shown in Table 1. The total accuracy of classification with a five-fold cross-validation was 56.5%.

**Table 1:** Confusion matrix from the test dataset showing the number and accuracy of assignments classified by the logistic regression into the two strategies (columns) versus the actual strategies (rows).

		Prediction		
		LFRR (56.2%)	RFLR (57.0%)	Total
Actuality	LFRR (59.6%)	300	204	504
	RFLR (53.5%)	234	270	504
	Total	534	474	1008

The percentages (in parentheses) represent the accuracies of row- and column-wise classification. The row-wise accuracy represents the percentage of correctly predicted assignments for the given strategies, while the column-wise accuracy represents the percentage of correctly predicted strategies, given the prediction.

First proposed by Vapnik^41^, based on the statistical learning technique, SVM can be used for pattern recognition and classification. The SVM approach has been shown to be an effective and robust method for classification, with desirable properties of scalability and efficiency to unobserved data. The confusion matrix of the SVM is shown in Table 2. The total accuracy of classification with a five-fold cross-validation was 68.8%.

**Table 2:** Confusion matrix from the test dataset showing the number and accuracy of assignments classified by the SVM into the two strategies (columns) versus the actual strategies (rows).

		Prediction		
		LFRR (68.0%)	RFLR (69.6%)	Total
Actuality	LFRR (70.9%)	357	147	504
	RFLR (66.7%)	168	336	504
	Total	525	483	1008

All modelling with dHMMs was conducted using a data-driven approach by maximizing the conditional likelihood. A five-fold cross-validation was used to determine the number of hidden states in the dHMM, of which different hidden-state configurations were *S* $\epsilon$ {2-2, 2-3, 3-3, 3-4, 4-4, 4-5, 5-5} corresponding to the number of hidden states used for modelling the LFRR and RFLR, respectively. The number of hidden states was determined by comparing the mean of accuracy of the validation sets. The increases in out-of-sample accuracies started to level off when the number of hidden states was {3-3}, suggesting that these are the optimal numbers of hidden states. The 6-state HMM achieved a classification accuracy of 76.8% with a 5-fold cross-validation for the test dataset. The confusion matrix of the dHMM is shown in Table 3.

**Table 3:** Confusion matrix from the test dataset showing the number and accuracy of assignments classified by the dHMM into the two strategies (columns) versus the actual strategies (rows).

		Prediction		
		LFRR (75.7%)	RFLR (77.7%)	Total
Actuality	LFRR (78.6%)	396	108	504
	RFLR (74.9%)	127	377	504
	Total	532	485	1008

Besides that, we also estimated HMM for each participant and tested if there was significant number of participants correspond to the 3-3-state HMM. We found that there were only five participants whose classification accuracies of 3-4-state HMM were higher than these of 3-3-state HMM. Furthermore, we additionally compared the paired accuracy values for 3-3-state and 3-4-state configurations with a Wilcoxon signed rank test. The difference between the 3-3-state and 3-4-state models of these five participants was not statistically significant (*p* = 0.095). Since the data did not support the preference of a 3-4-state model over a 3-3-state model, the less complex model should be preferred. Therefore, the model with {3-3} hidden states is further confirmed when using the majority vote-based model selection scheme^42^.

Ten times of five-cross-validation were conducted with the training dataset, and the series of accuracies were pooled across subjects and compared across cross-validation runs. The classification accuracies of any two classifiers among the three classifiers were all significant (Logistic vs. SVM: *F*(1,18) = 244.87, *p* = 6.338E-12; SVM vs. HMM: *F*(1,18) = 231.84, *p* = 1.003E-11; Logistic vs. HMM: *F*(1,18) = 1074.52, *p* = 1.672E-17), suggesting that the classification performance of HMM was significantly higher than that of other two classifiers.

### Interpretations of HMM parameters

This analysis was designed to determine exactly how the patterns of subjects’ fixations might reveal the cognitive processes underlying mental rotation. Properly interpreting the parameters of a discriminatively trained joint density model (e.g., a dHMM) remains an open question. From the work of Simola *et al*.^33^, we know that a straightforward way of interpreting parameters is to compare and report the values of parameters from ordinary and conditional maximum likelihood. In the current experiment, the dHMM parameters of the two mental-rotation strategies (Table 4) are roughly the same, suggesting that our model uses the eye-movement information containing the mental-rotation strategies fairly well.

**Table 4:** The dHMM parameter values for encoding and searching, comparison, and searching on one-side pair states for each task strategy.

		Encoding and searching	Comparison	Searching on one-side
Initial probability	LFRR	9%	5%	35%
	RFLR	15%	4%	31%
LFRR	FD (ms)	96 ± 3	170 ± 2	179 ± 2
	SL (px)	37 ± 4	567 ± 1	152 ± 2
	AOIs of fixations			
	AOI-1	44%	28%	18%
	AOI-2	14%	21%	18%
	AOI-3	29%	31%	39%
	AOI-4	13%	20%	26%
	Saccade direction			
	SD-1	89%	0%	56%
	SD-2	4%	1%	43%
	SD-3	5%	68%	0%
	SD-4	2%	31%	1%
RFLR	FD (ms)	108 ± 2	162 ± 2	172 ± 2
	SL (px)	33 ± 4	569 ± 1	159 ± 2
	AOIs of fixations			
	AOI-1	57%	32%	46%
	AOI-2	14%	21%	22%
	AOI-3	21%	30%	18%
	AOI-4	8%	17%	14%
	Saccade direction			
	SD-1	94%	2%	43%
	SD-2	3%	1%	49%
	SD-3	2%	66%	5%
	SD-4	1%	31%	3%

FD, fixation duration; SL, saccade length. The standard deviation *σ* is reported with respect to the mean *μ* by *μ* + *σ*.

The dHMM that best fit our data segmented the subjects’ cognitive processes under the LFRR and RFLR strategies into three states (Table 4).

The first set of processing states was labelled *encoding and searching* because the percentage of SD-1 saccade direction was approximately 90% in both tasks, which suggested that most saccades shifted within the current AOI. Moreover, in this process, we found that more fixations were distributed on the AOI-L1 and AOI-R1, suggesting that the subjects were inclined to perceptually encode and search the features and orientations of the AOI-L1 and AOI-R1, which are likely to be more critical and informative.

The second set of processing states was labelled *comparison* because the total percentage of SD-3 and SD-4 saccade direction was above 97% in both tasks, suggesting that most fixations shifted back and forth between the two figures in this process. Additionally, the percentage of SD-3 was above 65%, much higher than that of SD-4, suggesting that the subjects were inclined to compare the corresponding segments of the two figures.

With a combined probability of 66% (Table 4), the subjects began the assignments from the state which we termed *searching on one-side pair*, because the total percentage of SD-1 and SD-2 saccade direction was above 90% in both tasks, which suggested that the subjects were inclined to encode and search the information within one-side pair figure. Importantly, in this process, more fixations were distributed on the *right-side* pair (AOI-R1 and AOI-R2) in the LFRR strategy, whereas in the RFLR strategy, more fixations were distributed on the *left-side* pair (AOI-L1 and AOI-L2). The results indicated that in this process, subjects are inclined to search information on the right-side pair in the LFRR strategy and on the left-side pair in the RFLR strategy.

### Transitions between states

**Figure 3:**
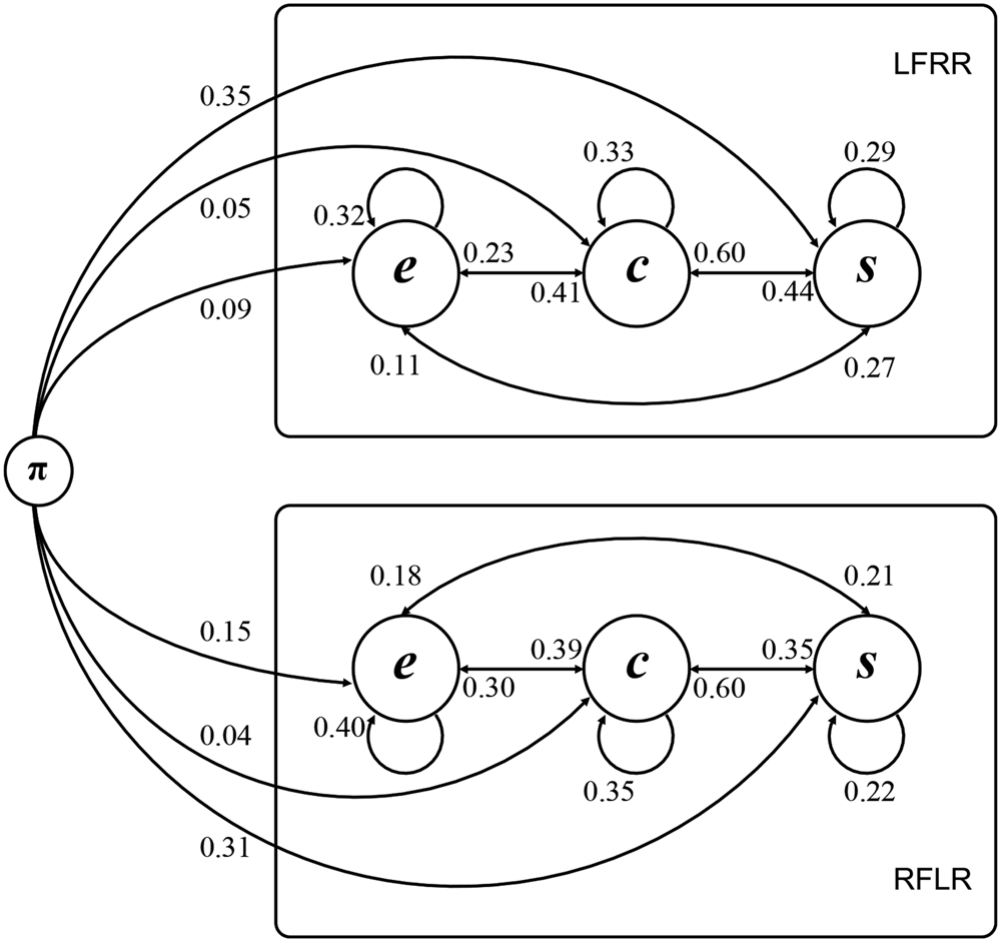
Topology of the transition probabilities in the dHMM. Hidden states are denoted by circles, and transitions among hidden states are denoted by arrows, along with their probabilities. The beginning of the sequence is denoted by π. The capital letters on the right denote the experimental sessions with different mental-rotation strategies (LFRR = left-side-fixed right-side-rotated, RFLR = right-side-fixed left-side-rotated), and the small letters within the circles denote the names of the hidden states or processes (*e* = *encoding and searching*, *c* = *comparison*, *s* = *searching on one-side pair*).

The transition probabilities of the dHMM are shown in Fig. 3. In the LFRR strategy, subjects switched more often from the process of *encoding and searching* (with 68% probability) than that in the RFLR strategy (with 60% probability), whereas the opposite was observed in the process of *searching on one-side pair* (71% probability in the LFRR vs. 78% probability in the RFLR). The probabilities for the transitions from the remaining states in the two strategies were similar, suggesting that the opposite mental-rotation strategies shared roughly the same cognitive processes.

### Processes and strategies in mental rotation

In mental-rotation tasks, subjects probably begin with *searching on one-side pair*, in which subjects have a high probability of searching on the right-side pair figure in the LFRR strategy and on the left-side pair figure in the RFLR strategy. The process of *searching on one-side pair* is the largest difference in cognitive processing between the two mental-rotation strategies. After the previous process, subjects are more likely to *compare* two figures. Subsequently, subjects have similar probabilities of switching from comparison to these three processing states (*encoding and searching*, *comparison*, *searching on one-side pair*), to which the probabilities of switching from the process of *encoding and searching* were similar. In summary, the series of three processing states occurred alternately, instead of sequentially.

## Discussion

This study correlated measurements of eye movements to mental-rotation tasks in an effort to uncover the processes of mental-rotation. Moreover, our experimental setup differed from that of traditional methods used to study mental rotation, where controlled experiments are designed to observe changes in eye movements (e.g., average fixation durations, number of fixations or saccade amplitudes) or behavioural results when cognitive processes are manipulated^3, 9, 15, 38, 43^. Instead, we designed a less-controlled experiment and used an advanced statistical modelling and data-driven approach with an HMM to make inferences regarding cognitive processing during mental rotation. Moreover, to the best of our knowledge, we are the first to instruct research subjects to perform mental rotation using different strategies of mentally rotating processes; that is, we are the first to distinguish between mental-rotation strategies and cognitive-processing patterns.

Our modelling results revealed how subjects shifted their gaze and cognitively processed as they proceeded with the mental-rotation tasks. The first set of processing states was termed *encoding and searching* because the rate of SD-1 (saccade within the same AOI) was the highest of the saccade direction types. Moreover, the probabilities of fixations in the AOI-L1 and AOI-R1 (the total probabilities were above 70%) were higher than those in the other two AOIs, suggesting that subjects preferred to perceptually encode and search the features of the Upper Arms (AOI-L1 and AOI-R1) of the stimulus figures. This process was characterized by shorter saccades and shorter fixations than the other two processes. Shorter saccades were understandable and reasonable because of the relatively small range of area within only one AOI. The shorter fixation durations in this process suggested that less attention and computational demands were required. In other words, perceptually encoding and searching the features of a stimulus required less cognitive-processing resources of the human brain.

The second set of states was labelled *comparison* because of the higher total probabilities of SD-3 (saccade to the corresponding AOI in the other figure) and SD-4 (saccade to the transformed AOI in the other figure) saccade directions and the uniform distribution of fixations in the four AOIs. In this process, subjects repeatedly looked back and forth between corresponding segments of the two stimulus figures. The features of this process were consistent with the prior proposal that the repeated fixation of corresponding segments was associated with the transformation and comparison process^3^.

We termed the third processing state *searching on one-side pair* because the total probability of SD-1 (saccade within the current AOI) and SD-2 (saccade to the other AOI in the same figure) was above 90%. Subjects typically began the process in mental-rotation tasks. Compared with the *encoding and searching* process, this process was characterized by longer saccades and longer fixations. Longer saccades were also understandable and reasonable because of the relatively longer distance between two AOIs than within one AOI. Both *comparison* and *searching on one-side pair* were characterized by longer fixation durations, suggesting higher computational demands and working-memory effects in these two processes than for *encoding and searching* ^3, 9, 44^. More importantly, this process exhibited significantly different fixation distributions in the AOIs between the two strategies (LFRR and RFLR). The subjects were inclined to search on the right-side pair figure in the LFRR task and the left-side pair in the RFLR task. These results suggested that when instructed to mentally rotate the right-side pair and keep the left-side pair fixed, subjects shifted more fixations on the right-side pair figure, whereas when instructed to mentally rotate the left-side pair and keep the right-side pair fixed, they shifted more fixations on the left-side pair figure. In summary, after encoding and memorizing the features and orientation of one-side pair figure, subjects shifted their gaze and attention to the other-side pair to search for corresponding features and orientation, then mentally rotated the corresponding segments, and finally compared the shape and orientation information and determined whether the objects matched or not.

A recent study identified various strategies in mental rotation, including holistic rotation, piecemeal rotation, and viewpoint-independent strategies^45^. In their classic study, Shepard and Metzler^1^ proposed that subjects used a holistic strategy that involves rotating the mental figure as a whole, similar to physical rotation. However, Shepard and Metzler^46^ also found that rotation was accomplished in a piecemeal fashion when subjects compared one rotated three-dimensional object with another. Furthermore, Just and Carpenter (1985) recorded eye movements while subjects compared 3D shapes and found that the pattern of fixation-shifts again suggested piecemeal rotation. These observations highlight the limits of the holistic nature of the representations that can be mentally rotated. Notably, our results were consistent with the previous suggestion that mental rotation is discrete process that is accomplished in a piecemeal fashion, being monitored after every rotation step to determine the target orientation^3, 4, 47–49^.

In addition, our model is able to predict mental-rotation strategies with an average accuracy of 76.8%, which is 26.8% units above pure chance (50% for two classes) and 20.3% units above the ground model (56.5% for logistic regression), which is very much in line with our initial expectation. First, the data set for the model contained all the data, including subjects with noisier eye-movement signals (subjects with low trial accuracy and inaccurate eye-tracking calibration were excluded from the analyses). Second, the tasks were not highly controlled. Instead, the subjects were permitted to freely choose their own processing strategies according to the instructions. However, some subjects may not have performed some of the trials using different cognitive strategies and patterns (*left-side-fixed right-side-rotated* or *right-side-fixed left-side-rotated*), which is the greatest limitation of our study. Mental rotation in those subjects who were instructed to perform alternative strategies that were unnatural or unfamiliar to them might have utilized different cognitive processes, which was not taken into consideration in our study. Third, the 300-Hz sampling rate of the Tobii Pro TX300 eye tracker quantized the gaze durations to 3-ms intervals. Using an eye tracker with a higher temporal and spatial resolution, the classification models may have the ability to predict the strategies more accurately because of the availability of more information. Furthermore, the coordinate systems have effects on recognition, information retrieval and spatial transformations, such as mental rotation^4^; thus, different subjects may employ different coordinate systems during mental rotation, which would also account for varying performance levels in mental-rotation tasks^4^. Alternative coordinate systems would explain some individual differences in different strategies in mental rotation. In addition, subjects processed some small-orientation figures (e.g., 0°, 30°) with shorter RTs and fewer fixation numbers (only one or two fixations in some trials), resulting in difficulty in conducting the training and prediction of models for the small-dimensional data and leading to the undesired accuracy of prediction to maintain the performance. This effect can also be demonstrated by the analysis of the higher accuracy of classification with consideration of data from large-orientation figures (e.g., 60°, 90°, 120°, 150°, 180°, etc.).

One limitation of this study is that subjects’ strategies were inferred from their reports and could not be determined objectively. Another limitation is the lack of consideration of gender differences. Further studies, such as combining eye movements and EEGs or fMRI, may provide more valuable functional information about the activities correlating with the cognitive processes reflected in eye-movement patterns and reveal additional differences in brain activation between the two mental-rotation strategies. Nevertheless, our eye-tracking study did confirm that there are differences in processing states between these two of mental-rotation strategies.

## Methods

### Subjects

Twenty healthy postgraduate student volunteers (all male to exclude the influence of gender factors), ranging from 24 to 30 years of age (26.2 ± 1.6 years), participated in the study at the Beijing Institute of Radiation Medicine. All subjects were right-handed with normal or corrected-to-normal vision, reported no history of neurological or psychological disorders, and were naive to the purpose and background of the study.

Each subject signed a written informed consent and received financial compensation regardless of performance. All methods were performed in accordance with the relevant guidelines and regulations and all experimental protocols were approved by the Ethics Committee of Beijing Institute of Radiation Medicine before the experiments.

### Stimuli

**Figure 4:**
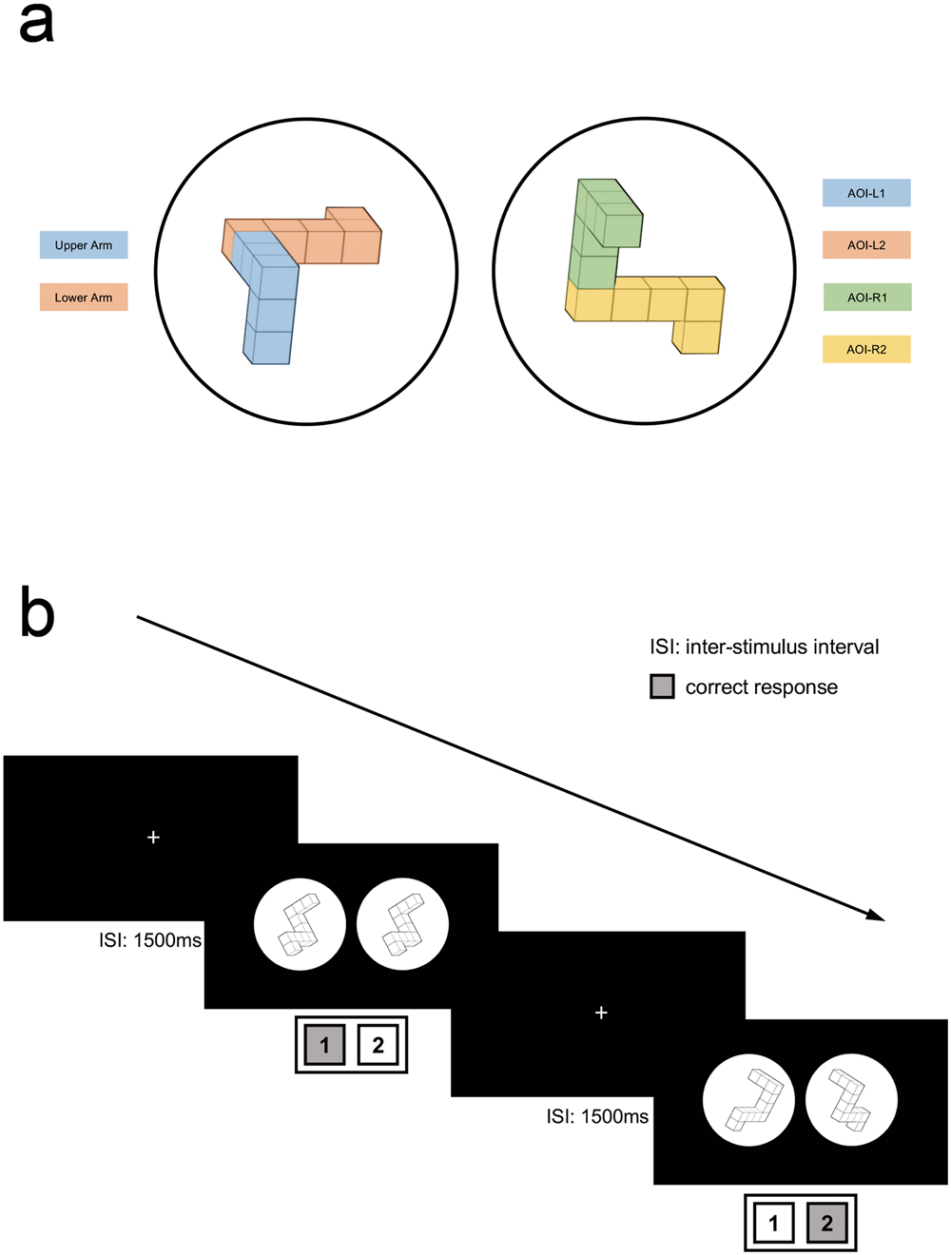
(**a**) The stimulus figure prototype of Shepard and Metzler’s mental-rotation task and the schematic depiction of the AOIs. The two orthogonal arms (blue) are labelled *Upper Arm*, while the two parallel arms (orange) are labelled *Lower Arm*. The Upper Arm and Lower Arm of the left figure are defined as AOI-L1 and AOI-L2, respectively, while the Upper Arm and Lower Arm of the right figure as AOI-R1 and AOI-R2, respectively. (**b**) Schematic diagram of the experimental procedure. The key in the gray panel indicates the correct response, with ‘1’ indicating a matched stimuli pair and ‘2’ indicating a mirrored stimulus pair.

The stimulus material was a subset of the original stimuli in the mental-rotation stimulus library created by Peters and Battista^50^, In which the stimulus pairs were comparable to those described by Shepard and Metzler^1^. We selected the third prototype in this library. By definition, the two orthogonal arms with a total of five cubes were labelled *Upper Arms*, while the two parallel arms were labelled *Lower Arms* (Fig. 4a, left-side).

Overall, this procedure yielded to 56 different stimuli: 7 angular disparities (0°, 30°, 60°, 90°, 120°, 150° and 180°) ×2 rotation axes (x-axis, z-axis) ×2 identities (identical, mirrored) ×2 symmetries (left-side and right-side shift, to exclude the influence of object locations). The centre-to-centre distance between the figures was 23.5 cm, and each figure was surrounded by a blank circle with a diameter of 21 cm.

To facilitate subsequent analysis, the Upper Arm and Lower Arm of the left-side figure were defined as AOI-L1 and AOI-L2, respectively, while the Upper Arm and Lower Arm of the right-side figure were defined as AOI-R1 and AOI-R2, respectively (Fig. 4a).

### Apparatus

The eye tracker was a Tobii Pro TX300 (developed by *Tobii Technology AB*, *Danderyd*, *Sweden*, bundled software*-*Tobii Pro Studio) with a 300-Hz sampling rate (binocular), a maximum total system latency of 10 ms and a spatial accuracy of 0.4°, corresponding to a distance of 65 cm between the eye tracker and the head^51^. The eye tracker was calibrated before the experiments using a set of 9 calibration points shown one at a time.

To ensure that all stimulus figures were viewed from a similar visual angle by all subjects, an optometrist’s viewing device was used to immobilize the subjects’ heads during the experiments. The use of the device maintained a distance of 65 cm between the monitor and the subjects’ heads, so each figure subtended approximately 18° of visual angle, and the centre-to-centre distance between the two figures subtended approximately 20°.

### Procedure

The current study contained three experimental sessions, which are described below.

Self mentally rotate (SMR): In experimental session 1, subjects were instructed to mentally rotate the two figures and determine whether the two figures were identical or not and then respond by pressing alternative buttons as quickly as possible with minimal errors, without any other restrictions. The SMR session was conducted but its data was not reported in the current manuscript because it has no effect on the final conclusions.

Left-side-fixed right-side-rotated (LFRR): In experimental session 2, in addition to the instructions in session 1, subjects were also instructed to mentally rotate only the right-side figure into alignment with the left-side figure and then determine whether the two figures were identical or not and respond.

Right-side-fixed left-side-rotated (RFLR): In experimental session 3, the instructions were the opposite of session 2, in which the subjects mentally rotated the left-side figure into alignment with the right-side figure.

All subjects participated in one experimental session per day and completed all experiments in a total of three days. Half of the randomly selected subjects were instructed to complete the experiments in the experimental order of session 1, session 2 and session 3, and the other half in the order of session 1, session 3 and session 2. This procedure was used to exclude the influence of session order and the interaction of sessions.

To reinforce the instructed strategies of the two latter experimental sessions before each formal experiment, the subjects were required to watch a video showing the left-side stationary and right-side rotating for session 2 and right-side stationary and left-side rotating for session 3. To introduce the subjects to the stimuli and the experimental task, practice sessions were conducted until the response accuracies of subjects reached at least 90%. During the formal experiment, subjects were asked to judge and determine whether the presented figures were identical or mirrored pairs using the instructed mental-rotation strategies and respond by pressing button ‘1’ for identical and pressing button ‘2’ for mirrored as quickly as possible. The maximum time of each stimulus presentation was limited to 8000 ms^9^. A white fixation crosshair was displayed during the 1500-ms inter-stimulus interval (ISI), and subjects were instructed to fixate on the cross until the onset of the trial (Fig. 4b).

### Modelling

In the experimental analysis, we used a *data-driven* approach, in which the data were used to determine the optimal parameters for different modelling issues. Five cross-validations with the training dataset were used to optimize the best model topology and parameters, and then the degree of fit was assessed with the test dataset to avoid model overfitting. Specifically, we employed the *supervised* classification methods, in which the operation contained both training and prediction stages. In the training stage, the dataset contained two segments: a set of *training* data values (the eye-movement features, e.g., fixation duration, saccade length) and the correct outcomes (the strategy labels of each subject, e.g., LFRR, RFLR). Then the classifier tuned the parameters of a classification model to minimize the error on the predicted versus the actual labels in the training dataset. In the prediction stage, the pre-tuned classification model was tested with a new dataset (test dataset), and the classification performance was measured by the error on the predicted versus the actual labels in the test dataset.

Goodness of the modelling configuration was measured in terms of both the total classification accuracy and the confusion matrix of prediction. Classification accuracy is the percentage of correctly predicted strategies divided by the total number of trials. The confusion matrix is a simplified formulation of receiver operating characteristic (ROC) curves, which are commonly used to compare classification methods to clearly and intuitively understand the trade-off between sensitivity (true positive rate) and specificity (1 – false positive rate).

In HMM algorithm, first, the observation at time *t* is assumed to be generated by some process where the state *S*_*t*_ is hidden from the observer. Second, given the value of *S*_*t−*1_, the current state *S*_*t*_ is independent of all the states prior to *t*−1; that is, the transition to the current state *S*_*t*_ depends only on the previous state *S*_*t−1*_. Pieters *et al*.^52^ showed that eye-movement behaviour follows this property. Additionally, *π*_*i*_, *i* = 1, …, *n* represents the probability of initiating the time sequence at state *S*_*i*_. Here, some states *j* may have *π*_*j*_ = 0, meaning that they cannot be initial states, and also $\sum _{i=1}^{n}{\pi }_{i}=1$. For time series *X*_*1*,*…*,*T*_ of observations, the full likelihood of the HMM is described as

1 $$P({X}_{1,\ldots ,T}|\lambda )=\sum _{\varphi }{\pi }_{1}P({X}_{1}|{S}_{1})\times \prod _{t=2}^{T}P({X}_{t}|{S}_{t})P({S}_{t}|{S}_{t-1})$$

where *φ* denotes all “paths” through the model, *S* ^*T*^ is the combination of hidden states for a sequence of length *T*, and *X*_*t*_ is the measured observation vector at time *t*.

In the algorithm related to the HMMs, three major problems must be solved: evaluation, decoding, and training. The algorithms used most commonly to solve these problems are called the Viterbi and Baum-Welch (BW) algorithms. The BW algorithm is a special case of the Expectation-Maximization (EM) algorithm, which can be proven to converge to a local optimum. Details about the algorithms are available in Rabiner^27^ and Huang *et al*.^53^.

Furthermore, as a generative model, the HMM can be converted to a discriminative model using Bayes formula to optimize the conditional likelihood of the model log*P*(*C*|*X*, *λ*). A dHMM is trained by assigning a set of actual hidden states *φ*_*c*_ corresponding to a certain class *c* and then for training data, maximizing the likelihood of the state sequences that go through the actual states. The parameters of a dHMM are optimized with a discriminative EM algorithm, which is a modification of the original BW algorithm.

### Feature extraction

#### Features for logistic regression and SVM

Logistic regression and SVM should use averaged features (or unidimensional variables) that can be derived from the eye-movement sequences, in which the contained information is similar to that in the HMM. Therefore, the features that we used are listed below:

i. Length of the eye-movement sequence (number of fixations).
ii. Mean of the fixation duration (in ms).
iii. Standard deviation of the fixation duration.
iv. Mean of the saccade length (in pixels).
v. Standard deviation of the saccade length.

#### Features for dHMM

We used four features of each fixation from the fixation-saccade data filtered with the raw eye-movement data. The features are listed below with the corresponding modelling distribution or indicator value shown in parentheses.

i. Logarithm of fixation duration in milliseconds (one-dimensional Gaussian).
ii. Logarithm of outgoing saccade length in pixels (one-dimensional Gaussian).
iii. Outgoing saccade direction (quantized to four defined different directions).

We conducted an HMM that evoked the fixation durations by changing the temporal scale of the HMM into fixation counts. Instead of conducting an HMM that is in state *s* for time *t*, …, *t* + *τ*, we conducted an HMM that is in state *s* for the *ith* fixation, which had the duration *τ*. We then modelled the logarithm of fixation durations with a Gaussian to simplify assumptions.

The saccade lengths (quantified as pixels) were computed between the gaze location at the beginning of the current fixation and the end of the next fixation.

The outgoing saccade direction (SD) from current fixation was encoded with an indicator variable containing four defined values:

SD-1 – saccade within the identical AOI (e.g., AOI-L1 to AOI-L1, AOI-L2 to AOI-L2, AOI-R1 to AOI-R1, and AOI-R2 to AOI-R2).

SD-2 – saccade to the other AOI in the identical figure (e.g., AOI-L1 to AOI-L2, AOI-L2 to AOI-L1, AOI-R1 to AOI-R2, and AOI-R2 to AOI-R1).

SD-3 – saccade to the corresponding AOI in the other figure (e.g., AOI-L1 to AOI-R1, AOI-L2 to AOI-R2, AOI-R1 to AOI-L1, and AOI-R2 to AOI-L2).

SD-4 – saccade to the transformed AOI in the other figure (e.g., AOI-L1 to AOI-R2, AOI-L2 to AOI-R1, AOI-R2 to AOI-L1, and AOI-R1 to AOI-L2).

### Statistical analysis

Two-way repeated measures analysis of variance (ANOVA) was conducted on the behavioural performance data, with accuracy and RT, with Strategy (two levels: LFRR and RFLR) as the between-subjects factor and Angular disparity (seven levels: 0°, 30°, 60°, 90°, 120°, 150° and 180°) as the within-subjects factor. The Huynh-Feldt correction was conducted when the assumptions of violation sphericity occurred, and partial eta-squared value was reported for effect size.

One-way ANOVA was conducted on the average RT for the two sessions (session 2 and session 3) and two groups (the two groups of subjects that completed the experiments with different experimental session orders) to determine the session order effect.

Ten times of five-cross-validation were conducted with the training dataset, and then ten classification accuracies with the test dataset were obtained for each classification methods. Then one-way ANOVA was conducted on the classification accuracies of any two classifiers among the three classifiers.

### Data availability

All data generated or analysed during this study are included in this published article.
